# Focally Spared Region in Diffuse Type of Liver Metastasis From Renal Cell Carcinoma

**DOI:** 10.1016/j.gastha.2021.08.003

**Published:** 2022-02-03

**Authors:** Tasuku Nakabori, Yutaro Abe, Keiichiro Honma, Kazuyoshi Ohkawa

**Affiliations:** 1 Department of Hepatobiliary and Pancreatic Oncology, Osaka International Cancer Institute, Osaka, Japan; 2 Department of Diagnostic Pathology and Cytology, Osaka International Cancer Institute, Osaka, Japan

**Keywords:** Liver Metastasis, Focally Spared Region, Regional Interface Hepatitis

## Abstract

Liver metastasis is not uncommon in various malignant tumors. Most of liver metastases present as discrete masses. However, liver metastases can appear as diffuse infiltrating neoplasms. Infiltration of malignant cells can provoke hepatic fibrosis, which mimics cirrhosis. Progress of diffuse type of liver metastasis remains unclear because of its difficulty in diagnosis or aggressive nature. In this report, we describe a 27-year-old woman with diffuse type of liver metastasis from renal cell carcinoma. The present case showed atypical findings of clinical images on liver examinations, which was histologically diagnosed as a focally spared region in diffuse type of liver metastasis by needle biopsy. Liver fibrosis was not observed in the biopsy specimen. Our case report suggests that liver biopsy is essential for diagnosis of diffuse type of liver metastasis, and the spared region can be observed in the metastatic process of the diffuse type.

## Introduction

Various cancers metastasize to liver via hematogenous or lymphatic route. Most liver metastases present as discrete masses. However, liver metastases can appear as diffuse infiltrating neoplasms that are not easily identified radiographically.^1^ Diffuse type of liver metastasis can progress rapidly, resulting in acute liver failure or cirrhosis.^2^ Therefore, progress of infiltration type of liver metastasis remains unknown.

The present report describes an unresectable advanced renal cell carcinoma patient with diffuse type of liver metastasis. Clinical images on liver examinations had atypical findings, which was histologically diagnosed as a focally spared region in diffuse type of liver metastasis. Liver fibrosis was not observed in the biopsy specimen. The findings suggest that the spared region can be observed in the process of the diffuse type of liver metastasis.

## Case Report

A 27-year-old woman with unresectable advanced renal cell carcinoma was introduced to our department with elevated hepatobiliary enzymes. The patient was taking axitinib, azelnidipine, and tramadol hydrochloride. She occasionally drank alcohol. Her laboratory tests showed elevated levels of aspartate aminotransferase (55 U/L; reference range, 13–30 U/L), alanine aminotransferase (33 U/L; reference range, 7–23 U/L), lactate dehydrogenase (326 U/L; reference range, 124–222 U/L), gamma-glutamyl transpeptidase (257 U/L; reference range, 9–32 U/L), and alkaline phosphatase (1028 U/L; reference range, 106–322) and a normal platelet count, prothrombin time-to-international normalized ratio, and total bilirubin. The patient had no history of viral hepatitis, steatohepatitis, autoimmune liver disease, or metabolic liver disease. The patient stopped drinking alcohol and taking axitinib; however, her hepatobiliary enzyme levels did not change. Abdominal ultrasonography revealed coarse and heterogeneous relatively hyperechoic hepatic parenchyma as a whole and an irregular isoechoic region from the hepatic hilum to the right dorsal side, coursing along the anterior segmental branch of the portal vein (Figure A). Contrast-enhanced computed tomography (CT) revealed enhancement in the liver except for the region corresponding to the isoechoic region in the arterial phase (Figure B). In the portal phase, contrast remained longer and the region corresponding to the isoechoic region was enhanced. Gadolinium-ethoxybenzyl diethylenetriamine–enhanced magnetic resonance imaging was performed. The findings identified by contrast-enhanced CT were also seen. The region corresponding to the relatively hyperechoic hepatic parenchyma was clearly hypointense compared with the hyperintensity in the region corresponding to the isoechoic region in the hepatobiliary phase (Figure C). Liver biopsy was performed targeting the relatively hyperechoic hepatic parenchyma and the isoechoic region. The microscopic findings were as follows. Coarse and heterogeneous relatively hyperechoic hepatic parenchyma, wherein scattered clusters of malignant cells positively stained for PAX8 (Figure D), were observed in Glisson's capsule, which occluded the interlobular vein (arrow), and inflammatory cells had infiltrated into the portal canal and destroyed the structure of the interlobular bile ducts (Figure E, hematoxylin and eosin stain, arrow head); and isoechoic region (Figure F, PAX8), in which malignant cells were rarely seen and only slight inflammatory change was present. The patient was finally diagnosed with liver metastasis from renal cell carcinoma, which was classified as progressive disease while undergoing axitinib according to the Response Evaluation Criteria in Solid Tumors (RECIST) criteria.^3^

**Figure:**
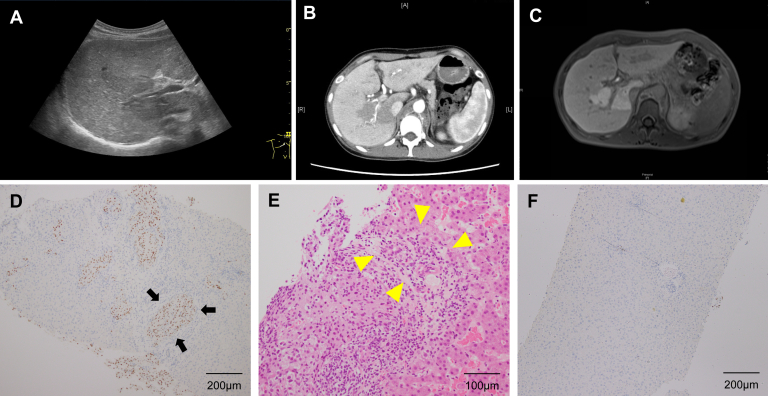
Abdominal ultrasonography revealed coarse and heterogeneous relatively hyperechoic hepatic parenchyma and an irregular isoechoic region (A). Contrast-enhanced CT revealed enhancement in the liver except for the region corresponding to the isoechoic region in the arterial phase (B). Gadolinium-ethoxybenzyl diethylenetriamine–enhanced magnetic resonance imaging revealed the region corresponding to the relatively hyperechoic hepatic parenchyma was clearly hypointense (C). Representative images of PAX8 immunohistochemical staining (D) and hematoxylin-eosin staining (E) in the coarse and heterogeneous relatively hyperechoic hepatic parenchyma. Arrows indicate PAX-8 positive cells. Arrowheads indicate the interlobular bile ducts destroyed by infiltration of inflammatory cells. Representative images of PAX8 immunohistochemical staining in the isoechoic region (F).

## Discussion

In this report, we present a patient with advanced renal cell carcinoma with elevated hepatobiliary enzymes. The present case showed atypical liver findings of clinical images, which was diagnosed as a focally spared region in diffuse type of liver metastasis by histological analysis of liver biopsy specimen.

Liver metastasis is not uncommon in renal cell carcinoma, as with various other malignant tumors.^3^ Although most of liver metastases present as discrete masses which was easily detected by imaging modalities, liver metastases can appear as diffuse neoplasms that are not easily identified radiographically.^1^ Infiltration of malignant cells can provoke hepatic fibrosis, which mimics cirrhosis.^4^ In the present case, histological analysis by liver biopsy led us to the correct diagnosis and revealed that the isoechoic region located in the anterior segment was focally spared from the invasion of renal cell carcinoma. The hepatic arterial flow in diffuse liver metastasis was presumed to be compensating for the attenuated portal flow in the peripheral region owing to tumor thrombosis. The elevated hepatobiliary enzymes were caused mainly by interlobular bile duct damage. The lack of liver fibrosis may indicate that the present case was in the early stage of the infiltration type of liver metastasis. As the liver metastasis progresses, the focally spared area may disappear and the patient might develop carcinomatous cirrhosis.

In summary, this report described a patient with diffuse type of liver metastasis with a focally spared lesion. Diffuse type of liver metastasis is not easily identified radiographically, and liver biopsy is essential for correct diagnosis. The spared region was observed in the metastatic process of diffuse type, and it was located from the hepatic hilum to the right dorsal side in the present case. As this is one brief report describing the spared region in diffuse type of liver metastasis, its frequent site remains unclear. Further accumulation of the cases will be required to elucidate it.
